# Supplementary material for: Cash transfers for HIV prevention: A systematic review
Source: PLoS Med. 2021 Nov 29;18(11):e1003866. doi: 10.1371/journal.pmed.1003866 (PMC8668130; doi:10.1371/journal.pmed.1003866)
Supplement: S1 Text — (DOCX) [file pmed.1003866.s004.docx]

**Pubmed:**

(((cash[tiab] OR "cash incentive" OR "cash incentives" OR "cash transfer" OR "cash transfers" OR "financial incentive" OR "financial incentives" OR "cash reward" OR "cash rewards" OR "monetary reward" OR "monetary rewards" OR "contingency management" OR savings[tiab] OR scholarship*[tiab] OR "school uniform" OR "school fee" OR "school fees" OR "uniform cost" OR "uniform costs")) AND ( "2000/01/01"[PDat] : "3000/12/31"[PDat] ))) AND (((hiv[tiab] OR hiv infections[mesh] OR sexual partners[mesh] OR "sexual partner" OR "sexual partners" OR "sexual behavior" OR "sexual behaviour" OR sexual behavior[mesh] OR condoms[mesh] OR condom*[tiab] OR sexually transmitted diseases[mesh] OR std[tiab] OR stds[tiab] OR sti[tiab] OR stis[tiab] OR "transactional sex" OR "sexual debut")))

**PsycInfo:**

(((cash.ti,ab OR cash incentive OR cash incentives OR cash transfer OR cash transfers OR financial incentive OR financial incentives OR cash reward OR cash rewards OR monetary reward OR monetary rewards OR contingency management OR savings.ti,ab OR scholarship*.ti,ab OR school uniform OR school fee OR school fees OR uniform cost OR uniform costs)) AND (((hiv.ti,ab OR "hiv infections" OR "sexual partners" OR sexual partner OR sexual partners OR sexual behavior OR sexual behaviour OR "sexual behavior" OR condoms OR condom*.ti,ab OR "sexually transmitted diseases" OR std.ti,ab OR stds.ti,ab OR sti.ti,ab OR stis.ti,ab OR transactional sex OR sexual debut))))

**Web of Science:**

TOPIC: (cash OR "cash incentive" OR "cash incentives" OR "cash transfer" OR "cash transfers" OR "financial incentive" OR "financial incentives" OR "cash reward" OR "cash rewards" OR "monetary reward" OR "monetary rewards" OR "contingency management" OR savings OR scholarship* OR "school uniform" OR "school fee" OR "school fees" OR "uniform cost" OR "uniform costs") AND (hiv OR "hiv infections" OR "sexual partners" OR "sexual partner" OR "sexual partners" OR "sexual behavior" OR "sexual behaviour" OR "sexual behavior" OR condoms OR condom* OR "sexually transmitted diseases" OR std OR stds OR sti OR stis OR "transactional sex" OR "sexual debut")

Refined by: PUBLICATION YEARS: ( 2019 OR 2011 OR 2004 OR 2018 OR 2010 OR 2003 OR 2017 OR 2009 OR 2002 OR 2016 OR 2008 OR 2001 OR 2015 OR 2007 OR 2000 OR 2014 OR 2006 OR 2013 OR 2005 OR 2012 )

**EconLit:**

(((cash OR "cash incentive" OR "cash incentives" OR "cash transfer" OR "cash transfers" OR "financial incentive" OR "financial incentives" OR "cash reward" OR "cash rewards" OR "monetary reward" OR "monetary rewards" OR "contingency management" OR savings OR scholarship* OR "school uniform" OR "school fee" OR "school fees" OR "uniform cost" OR "uniform costs")) AND (((hiv OR "hiv infections" OR "sexual partners" OR "sexual partner" OR "sexual partners" OR "sexual behavior" OR "sexual behaviour" OR "sexual behavior" OR condoms OR condom* OR "sexually transmitted diseases" OR std OR stds OR sti OR stis OR "transactional sex" OR "sexual debut"))))
