# Supplementary material for: Cash transfers for HIV prevention: A systematic review
Source: PLoS Med. 2021 Nov 29;18(11):e1003866. doi: 10.1371/journal.pmed.1003866 (PMC8668130; doi:10.1371/journal.pmed.1003866)
Supplement: S2 Text — (PDF) [file pmed.1003866.s005.pdf]

1. Background/Purpose

Globally, cash transfers have become one of the most popular policy strategies to achieve social protection and development goals in a number of different domains. Programs that provide non-contributory cash payments now reach over one billion people across more than 130 countries. Evidence from these programs consistently points to their positive impacts on monetary poverty, education, health and nutrition, productivity and employment, and empowerment. Alongside large, national programs, there are a range of other initiatives and interventions that have utilized cash payments to achieve specific outcomes, many of them either education or health based. Given the success of cash transfers in improving health outcomes and addressing upstream drivers of HIV risk such as poverty and education, there has been an increasing interest in the potential of cash transfers to improve HIV prevention and care outcomes for the last decade. Cash transfers have also been incorporated into the prevention landscape in a number of UN approaches and targets. Most prevention strategies envision cash transfers and 'social protection' as part of a larger prevention package addressing uptake of prevention services and reduction of risk behavior. For this review, we focus on both cash transfer programs and other cash assistance models to achieve HIV prevention.

Recently, there has been a push to synthesize evidence on the impacts of cash transfers across the wide range of outcomes including child and adolescent development, consumption of temptation goods, partner violence, and the social determinants of health. Recent reviews have also documented the impacts of structural interventions on HIV prevention for young women and household economic strengthening for HIV outcomes, but broader evidence on cash transfer programs on HIV prevention has not been systematically reviewed for almost a decade. The aim of this review is to examine current evidence related to cash for HIV prevention in order to provide an updated synthesis of impacts and help inform programmatic decision-making.

2. Objectives/Review Question

Our team reviewed the impact of cash transfers on HIV prevention in 2012. The aim of this review is to examine current evidence related to cash for HIV prevention in order to provide an updated synthesis of impacts and help inform programmatic decision-making.

To update the systematic review of the published literature on the effects of cash transfers (and related cash assistance programs) on HIV prevention (HIV-negative populations), to include evidence of impact on:

- HIV infection and related STIs
- Sexual risk behavior

3. Methods

Overview criteria for studies:

1) analyzed either cash transfer programs, savings program, or programs to reduce school costs, and
2) reported impacts on HIV and HIV prevention related outcomes including HIV infection (incidence and prevalence), other sexually transmitted infections (STIs) (all infections; incident or prevalent), and sexual risk behavior outcomes including condom use, sexual debut, number of partners, transactional sex, older partners, and other related behavioral outcomes

Search Criteria:

Type of intervention:

- Cash transfer interventions to alleviate poverty including government social protection programs for families and individual structural cash transfer interventions, and individual incentive-based cash transfer programs (e.g. contingency management)

- Interventions that have a cash component including programs to reduce school costs and matched savings programs

Type of outcome:

1. Primary
    - HIV incidence/ prevalence
2. Secondary
    - STI incidence/ prevalence
    - Sexual behavior: condom use
    - Sexual behavior: sexual debut
    - Sexual behavior: numbers of partners
    - Sexual behavior: older partners
    - Sexual behavior: transaction sex
    - Sexual behavior: combined behavioral risk scores

Populations:

- HIV-negative populations for which cash transfer interventions are targeted

Type of studies:

- quantitative studies of experimental (RCTs) and nonexperimental evaluations of cash transfer and closely related (i.e., interventions to reduce school costs, and matched savings programs) programs. No restriction of studies by type of effect that was reported (e.g. Odds ratio (OR)).

Type of publication:

- published and gray literature

Publication Dates:

- 2000 – Present (July 2019)

Search strategy:

- Search keywords: (cash transfer, cash incentive, financial incentive, cash reward, monetary reward, contingency management, savings, scholarship, school uniform or school fee) and (HIV, STD, STI, condom use, sexual debut, number of partners, transactional sex, older partner or sexual behavior).
- Databases: Google Scholar, Psych Info, Econlit, PubMed, Scopus
- Informal Search: JPAL, Innovations for Poverty Action

4. Data Collection

Studies will be searched for the databases Psych Info, Econlit, and PubMed using the keywords. Study abstracts returned from the search will be imported from each database and combined into Covidence online software. Two reviewers will independently screen the abstracts using the search criteria and then examine the full text of selected articles for fit. Full-text articles will also be examined by both reviewers when discrepancies between reviewers arise during the abstract review. Disagreements will be reconciled through data extraction of search criteria. Inclusion will require the agreement of both reviewers. Grey literature will be sourced by contacting experts in the field and from other systematic reviews or commentaries on the topic. Data extracted from all selected studies include the study population, time frame, research design, and location, the type of intervention, program conditionalities, and effect sizes and significance for each of the relevant outcomes reported.
