# Supplementary material for: Cash transfers for HIV prevention: A systematic review
Source: PLoS Med. 2021 Nov 29;18(11):e1003866. doi: 10.1371/journal.pmed.1003866 (PMC8668130; doi:10.1371/journal.pmed.1003866)
Supplement: Attachment — Submitted filename: stoner.pdf [file pmed.1003866.s007.pdf]

This paper presents a systematic review of studies of HIV prevention.

I was asked for a statistical report and I interpret that to include all aspects of the design and conduct of the study. I see that this has been reviewed before but I have not seen it previously and have not studied any previous reviews.

Points of detail

Page 6 What was the search strategy and search terms for at least one database?

Page 6 For a condition which, sadly, has much impact in low– and middle–income countries a wider search might be helpful. Various empirical studies have indicated that the main bibliographic databases have varied and incomplete coverage of material not in English (Pilkington et al., 2005), and have limited coverage of material from low and middle–income country journals (Kieling et al., 2009; Syed Sheriff et al., 2008). An extension to some of the databases directed at global health would be helpful.

Page 7 Who did the extraction, was it done in duplicate, how was agreement checked?

Page 7 Was any language restriction applied? If it was then the message I draw from Grégoire et al. (1995); Moher et al. (1996); Egger et al. (1997); Jüni et al. (2002) is that bias is likely although it is hard to predict in advance the extent or direction of it. I would not necessarily commit the authors to translate everything but at least we should know what we are missing.

Points of more substance

Found no effect

The authors make repeated use of the phrase 'found no effect' starting in the abstract. What the classical hypothesis testing framework gives us is the ability to state no evidence of effect not to categorically rule out an effect. As it stands the authors would, for instance, report OR = 1.221 (95% confidence interval 1.002 to 1.489) in the text but would ignore 1.221 (0.998 to 1.495) as 'found no effect'.

Level of detail

The authors would benefit from re–reading the explanatory document for the PRISMA guidelines (Liberati et al., 2009) to get a picture of how much detail in reporting is suggested. I have mentioned a few examples above but really almost all the the items in their checklist would benefit from more reporting and if I tried to list them all my review would turn into a pastiche of Liberati et al. (2009).

Meta–analysis

It might have been possible to perform some form of meta–analysis but the presentation of results makes it impossible to say what could have been done. The authors state on page 7 that they extracted effect sizes and significance levels but these do not seem to be reported anywhere. Table 2 is suitable for a slide in a conference presentation but for a journal article we need the numbers.

Summary

Much still to do.

Michael Dewey

References

M Egger, T Zellweger-Zähner, M Schneider, C Junker, C Lengeler, and G Antes. Language bias in randomised controlled trials published in English and German. *Lancet*, 350:326–329, 1997.

G Grégoire, F Derderian, and J Le Lorier. Selecting the language of the publications included in a meta-analysis: is there a tower of Babel bias? *Journal of Clinical Epidemiology*, 48(1):159–163, 1995.

P Jüni, F Holenstein, J Sterne, C Bartlett, and M Egger. Direction and impact of language bias in meta–analyses of controlled trials: empirical study. *International Jounal of Epidemiology*, 31:115–123, 2002.

C Kieling, H Herrman, V Patel, and J J Mari. Indexation of psychiatric journals from low– and middle–income countries: a survey and a case study. *World Psychiatry*, 8:40–44, 2009.

A Liberati, D G Altman, J Tetzlaff, C Munroe, P C Gøtzsche, J P A Ioannidis, M Clarke, P J Devereaux, J Kleijnen, and D Moher. The PRISMA

statement for reporting systematic reviews and meta–analyses of studies that evaluate health care interventions: explanation and elaboration. *PLOS Medicine*, 6(e1000100), 2009.

D Moher, P Fortin, A R Jadad, P Jüni, T Klassen, J Le Lorier, A Liberati, K Linde, and A Penna. Completeness of reporting of trials published in languages other than English: implications for conduct and reporting of systematic reviews. *Lancet*, 347:363–366, 1996.

K Pilkington, A Boshnakova, M Clarke, and J Richardson. 'No language restrictions' in database searches: what does this really mean? *The Journal of Alternative and Complementary Medicine*, 11:205–207, 2005.

R J Syed Sheriff, C E Adams, P Tharyan, M Jayaram, L Duley, and the PRACTIHC Mental Health Group. Randomised trials relevant to mental health conducted in low and middle–income countries: a survey. *BMC Psychiatry*, 8(69), 2008.
