# Supplementary material for: Cash transfers for HIV prevention: A systematic review
Source: PLoS Med. 2021 Nov 29;18(11):e1003866. doi: 10.1371/journal.pmed.1003866 (PMC8668130; doi:10.1371/journal.pmed.1003866)
Supplement: Attachment — Submitted filename: Reviewers comments and RESPONSES_1.7.21.docx [file pmed.1003866.s006.docx]

**Reviewers comments and RESPONSES**

**Reviewer 1:**

1.1 “*This article will have a medium-degree of impact. While the article does make mention of some other outcomes evaluated (such as school enrollment and health service utilization), the outcomes reported on in this systematic literature review seem limited. It misses some of the key outcomes evaluated by studies importance to HIV/STI prevention, such as ART adherence, given that adherence impacts community viral load and HIV testing patterns. While some of these studies are captured in the Bastagli et al review, some are missed.”*

**RESPONSE: We did not look at ART adherence as we decided to limit this review to outcomes in HIV negative individuals as the focus was HIV prevention. The Bastagli review is focused on household diets and child health indicators as measures of health. While they do examine condom use and number of sexual partners in the domain of empowerment, they do not include any other HIV related outcome from our study.**

1.2 *“The narrative review of the findings is well reported. The introduction could flow better if the definition section on conditional vs. unconditional cash transfers came sooner and the section ended with the aim of the systematic review.*

**RESPONSE: We have now moved the aim of the systematic review to the end of the introduction. Additionally, the information on cash transfer has been moved up in the introduction and a new subheading has been added later related to the current evidence on cash transfers for HIV prevention.**

*1.3. In the results and discussion, it would be helpful to the reader if follow-up times for each study are reported and compared and contrasted. This is particularly important for studies that evaluated the effect of cash transfers on measures of disease incidence and prevalence.*

**RESPONSE: Follow up time has now been added as another characteristics in table 1. We also include a discussion of long-term impacts within the results section.**

*1.4 “The outcomes table is difficult to read. It is advised that the authors consider synthesizing this information into a figure that displays important key findings.”*

**RESPONSE: We have kept the table as is because we have not been able to find a graphic that would display the information in a more succinct way given the number of studies and outcomes. We would be open to adjusting the table (or creating a figure) to be more clear if there are specific aspects that are difficult to pull from the table. We have edited the table to try to make it clearer.**

*1.5 The last paragraph of the discussion presents quite a lot of information that has already been reported. It is advised that the authors consider making the concluding paragraph a discussion on why this matters for policy-makers and suggest a way forward for program planners that may be considering the implementation of cash transfers.”*

**RESPONSE: The last paragraph has been edited to remove redundant information. The last sentence now reads “Program and policy-makers interested in HIV prevention should consider programs that directly incentivize school enrollment and attendance or are conditional on attendance may have the largest impact on HIV risk for girls, especially in contexts where secondary school attendance is low.”**

*1.6 “The article is original, but it also builds on a previous review by Bastagli et al. With understanding that there are many outcomes measured and with differences in measurement, was there any consideration given to how outcomes may be quantitatively meta-analyzed? This would add strength and greater originality to the manuscript.”*

**RESPONSE: While a meta-analysis could be a useful exercise for some of the outcomes used in our study, our intention was to update the literature with the multitude of newly published studies and so we focused on many outcomes to capture the impacts of these programs on HIV prevention more broadly. We also examined a variety of types of cash programs in many different populations, making a meta-analysis difficult. Our review is distinct from the Bastagli et al. review we focus more broadly on cash transfer or incentive programs as compared to primarily national cash transfer programs and we compare study impacts on specific HIV-related outcomes.**

**Requests from the editors:**

2.1 “*Please revisit the title, removing the rhetorical question and placing the study descriptor ("A systematic review") after a colon.”*

**RESPONSE: The title has been edited to “Cash Transfers for HIV Prevention: A Systematic Review”**

2.2 “*Please remove information on funding and competing interests from the title page. In the event of publication, this information will appear in the article metadata, via entries in the submission form.”*

**RESPONSE: This information has been removed from the title page**

2.3 *“Please adapt the abstract to a three-part format. The final sentence of the "Methods and findings" subsection should begin "**Study limitations include ..." or similar and quote 2-3 of the study's main limitations.*”

**RESPONSE: The abstract is now divided into background, methods and findings, and conclusions. The following sentence has been added at the end of the abstract: “Study limitations include the small number of studies in key populations and examining interventions to reduce school costs and matched saving programs.”**

2.4 “*In the abstract, please note the countries or regions where the constituent studies were done; and state the proportions of different study designs included.”*

**RESPONSE:** **The following two sentences were added to the abstract: “Most studies were conducted in sub-Saharan African countries (N=21; South Africa, Tanzania, Malawi, Lesotho, Kenya, Uganda, Zimbabwe, Zambia, eSwatini) followed by Mexico (N=2), the United States (N=1), and Mongolia (N=1)). Of the 25 studies, 72% (N=18) were randomized trials, 20% (N=5) were observational, 4% (N=1) was a case control and 4% (N=1) was quasi experimental.”**

2.5 “*After the abstract, we will need to ask you to add an "author summary" section in non-identical prose. You may find it helpful to consult one or two recent research papers in PLOS Medicine to get a sense of the preferred style.*”

**RESPONSE: An author summary has now been added in the page after the abstract.**

2.6 “*Please add a new final sentence to the "Introduction" section of the main text, stating the study's aim.*”

**RESPONSE: The study aim has now been moved to be the final sentence in the introduction.**

*2.7. “Please quote the date of the search(es) in the Methods section.”*

**RESPONSE: The following sentence has been added to the methods section on page 6. “Searches where done on July 25th, 2019 and** **December X, 2020.**

*2.8 “Please update the literature search to a date in the past 3 months.”*

**RESPONSE: The literature search was updated as of December 17, 2020 and the entire manuscript has been updated accordingly.**  *2.9 “In the results section and any other instances, please make that "sub-Saharan Africa".*

**RESPONSE: This has been changed throughout the manuscript.** *2.10 “Throughout the paper, please take care in using words such as "effect" which imply causation. We generally restrict use of this language to describe evidence from randomized studies, and suggest alternatives such as "was associated with" or "impact" to describe findings from observational studies.”*

**RESPONSE: We have changed the term “effect” to “impact” or “association” throughout the manuscript, except when referring to a randomized trial.**

*2.11. “Throughout the text, reference call-outs should be in the form of numbers formatted as follows: "... 130 countries [1].".*

**RESPONSE: The reference call outs have been changed throughout the manuscript according to Vancouver format.**

*2.12 “Please reformat the reference list so that entries appear in order of citation in the text. Citations should be in Vancouver format, and all italics should be converted into plain text.”*

**RESPONSE: The references have been changed throughout the manuscript according to Vancouver format.** *2.13 “Please adapt the attached PRISMA checklist so that individual items are referred to by section (e.g., "Methods") and paragraph number rather than by line or page numbers, as the latter generally change in the event of publication. Please refer to the checklist in the methods section ("See S1_PRISMA_Checklist" or similar).”*

**RESPONSE: A reference to the PRISMA checklist has been added on page 6, paragraph 1 of the methods. The PRISMA checklist has now been revised so that each item includes the section and the paragraph number.**  *2.14 “Please also refer to the attached protocol document in the methods section and note when this was prepared. Was this registered in any publicly-accessible database?”*

**RESPONSE:** **The study protocol was prepared on February 4^th^ for UNAIDS but was not registered in any publicly accessible database. This has been added to the methods section on page 6.**
