# Supplementary material for: Cash transfers for HIV prevention: A systematic review
Source: PLoS Med. 2021 Nov 29;18(11):e1003866. doi: 10.1371/journal.pmed.1003866 (PMC8668130; doi:10.1371/journal.pmed.1003866)
Supplement: Attachment — Submitted filename: Reviewers comments and RESPONSES_5.4.21.docx [file pmed.1003866.s008.docx]

**Reviewer comments and RESPONSES**

**Requests from the editors:**

1.1. *Please resubmit your paper as a research article.*

**RESPONSE: Thank you. We will resubmit as a research article.**

1.2. “*In your abstract, please quote the date of the most recent search rather than "the present".”*

**RESPONSE: “The present” has been changed to December 17, 2020.**

1.3. “*In the Methods and Findings" subsection of your abstract, please adapt the presentation to the form: "... 20 (72%) were randomized trials ..."; and make that "... was a case-control study". Please make similar changes in the Main text.”*

**RESPONSE: The sentence was changed in the abstract and the main text to the following “Of the 27 studies, 20 (72% ) were randomized trials, 5 (20%) were observational, 1 (4%) was a case control and 1 (4%)) was quasi experimental.”**

*1.4 “At line 110, for example, please adapt the text to "...* *young women aged 13-22 years, and their families, to stay ...".”*

**RESPONSE: The sentence was changed as follows “a cash transfer intervention provided to young women aged 13-22 years, and their families, to stay to stay in school in Malawi found a reduction in HIV prevalence.”**

*1.5. “At line 166, for example, please quote 95% CI if available; similarly, if available please quote exact p values or "p<0.001".”*

**RESPONSE: Thanks, we have included 95% Cis or p-values if available.**

*1.6. “At line 221, please make that "... for girls than boys".”*

**RESPONSE: This sentence has been edited to “for girls than boys.”**
 *1.7. “Please hyphenate "quasi-experimental"; however "syphilis" should not take an initial capital.”*

**RESPONSE: Quasi-experimental has been hyphenated throughout and syphilis is now not capitalized.**

*1.8. “Please italicize species names.”*

**RESPONSE: Species names are now italicized.** *1.9. “Throughout the text, please style reference call-outs as follows: " ... STI negative [2,3].".”*

**REPONSE: Reference call outs have been changed throughout the manuscript.** *1.10. “Noting reference 9 and others, please ensure that all references have full access information.”*

**RESPONSE: Reference 9 has been corrected and other references have been checked for missing information.**

*1.11 “Please use the abbreviation "PLoS ONE".”*

**RESPONSE: The abbreviation has been changed to PLoS ONE** *1.12 “Please include the "studies included" in the reference list (we think that some are already included but not all).”*

**RESPONSE: All references have now been added to the manuscript.**

*1.13. “Please rename the PRISMA attachment "**S1_PRISMA_Checklist" or similar, and refer to it as such in the Methods section.”*

**RESPONSE: The PRISMA checklist has been renamed and is referred to in the methods.** *1.14. “You may wish to use the updated PRISMA, which has been published recently.”*

**RESPONSE: Thanks for making us aware of the new checklist. We are now using this updated checklist.**

**Reviewer #2:**

*2.1. “Page 6 What was the search strategy and search terms for at least one database?”*

**RESPONSE: Thank you, we have now added a third appendix with the exact search terms used for each database.**

*2.2. “Page 6 For a condition which, sadly, has much impact in low- and middle-income countries a wider search might be helpful. Various empirical studies have indicated that the main bibliographic databases have varied and incomplete coverage of material not in English (Pilkington et al., 2005), and have limited coverage of material from low- and middle-income country journals (Kieling et al., 2009; Syed Sheri_ et al., 2008). An extension to some of the databases directed at global health would be helpful.”*

**RESPONSE: This is a good point. We ensured that studies that we knew of from our prior review on the topic were captured in the four main databases and were within the fields that publish these types of articles including economics and public health. To capture additional studies, we searched grey literature, spoke with experts, and reviewed all studies reported in other systematic reviews on this topic. Therefore, we believe that our search was able to adequately capture studies in this area. We have added a limitation that we did not include studies not in English.**

*2.3. “Page 7 Who did the extraction, was it done in duplicate, how was agreement checked?”*

**RESPONSE: Details about how the data were reviewed are now provided in the methods section. We have specified that the two reviewers who did the review were MCDS and KK, who extracted the data. A sentence has been added to state that “Discrepancies were reviewed again by the two reviewers to come to a joint decision.”**

*2.4. “Page 7 Was any language restriction applied? If it was then the message I draw from Gregoire et al. (1995); Moher et al. (1996); Egger et al. (1997); Juni et al. (2002) is that bias is likely although it is hard to predict in advance the extent or direction of it. I would not necessarily commit the authors to translate everything but at least we should know what we are missing.”*

**RESPONSE: Thank you for this point. We have now added that English language was a criterion for inclusion. We were unable to review studies not in English as we don’t have this expertise on our team. The following sentence has been added as a limitation of the study: “we did not include studies that were not in English and may be missing additional studies not published in English.”**

*2.5. “The authors make repeated use of the phrase `found no effect' starting in the abstract. What the classical hypothesis testing framework gives us is the ability to state no evidence of effect not to categorically rule out an effect. As it stands the authors would, for instance, report OR = 1.221 (95% confidence interval 1.002 to 1.489) in the text but would ignore 1.221 (0.998 to 1.495) as `found no effect'.”*

**RESPONSE: Thanks for this point. We understand this concern and have tried to revise the wording and add in effect estimates throughout the document. We were unable to do a meta-analysis due to the number of outcomes and types of cash transfers assessed. Almost all of these studies were RCTs with large sample sizes. More extensive details on the studies are included in appendix 1. We have also now included a limitation about this point. “Given that were we unable to do a meta-analysis, studies are summarized by statistical significance and should also be considered with the estimates in appendix 1.”**

*2.6. “The authors would benefit from re-reading the explanatory document for the PRISMA guidelines (Liberati et al., 2009) to get a picture of how much detail in reporting is suggested. I have mentioned a few examples above but really almost all the items in their checklist would benefit from more reporting and if I tried to list them all my review would turn into a pastiche of Liberati et al. (2009).”*

**RESPONSE: Additional detail has been added for the points that were mentioned and others. We have also included the PRISMA checklist as an appendix with details on where in the manuscript each point is included.**

*2.7. “It might have been possible to perform some form of meta-analysis but the presentation of results makes it impossible to say what could have been done. The authors state on page 7 that they extracted effect sizes and significance levels but these do not seem to be reported anywhere. Table 2 is suitable*

*for a slide in a conference presentation but for a journal article we need the numbers.”*

**RESPONSE: All effect estimates are shown in appendix 1 but are not presented in table 2 to make the information easier to interpret quickly. The following sentence was added to the methods section: “We did not do a meta-analysis given the range of outcomes and types of interventions assessed.”**

**Reviewer #3:**

3.1. “*The authors may want to consider changing the title. The scope of the review covers more than just HIV prevention and several of the studies do not assess HIV outcomes.”*

**RESPONSE: We have kept the title as is because we consider sexual risk behaviors to also be a means of HIV prevention.**

3.2. “*Page 6, line 55 - there is a superscript "h" in the middle of the sentence 3.*”

**REPONSE: The superscript h has been deleted.**

3.3 “P*age 9, line 127 - define HSV-2 at first use. It is written in full in line 139.”*

**RESPONSE: HSV-2 has now been defined on line 130.**

3.4. “*Page 10, line 155, 156, 159 - there are a couple of extra brackets.”*

**RESPONSE: The extra brackets have been deleted.**

3.5. “*Page 11, line 175 - the Minnis et al ref needs to be provided in Vancouver style.”*

**RESPONSE: The Minnis reference has been correct to Vancouver style.**

3.6. “*Page 11, line 176 - the one bracket "(" after the references (25-29) needs to be removed.”*

**RESPONSE: The bracket has now been removed.**

3.7. *“The point about the limited data in key populations is made several times in the discussion, e.g. page 14, line 248, page 15, line 273 and 276 - some of the redundancy can be removed.”*

**RESPONSE: The point about key populations has been deleted on line 276.**

3.8. *References - There is some inconsistency with the formatting of the references. Ref 2 for example, ref 9 is missing volume and page numbers, provide url for ref 27 if available, ref 39 has "Suppl" three times*

**RESPONSE: Thanks for catching these issues. Reference 1, 9, 27, and 39 have been corrected.**

3.9*. “Table 2 - this table contains a lot of information and is not easy to follow. Not all outcomes are assessed in each study. Perhaps a not assessed (NA) can be added to the cells where the data are not assessed*.”

**RESPONSE: A dash has now been added to cells that were not assessed. We decided not to use NA because the effects became harder to see.**
