# Supplementary material for: Cash transfers for HIV prevention: A systematic review
Source: PLoS Med. 2021 Nov 29;18(11):e1003866. doi: 10.1371/journal.pmed.1003866 (PMC8668130; doi:10.1371/journal.pmed.1003866)
Supplement: Attachment — Submitted filename: Responses to editor and reviewer-10.28.21.docx [file pmed.1003866.s009.docx]

**Response to editors and reviewer:**

**Requests from Editors:**

1.1 *Noting referee 2's comments, we ask you to do an additional search using this methodology and incorporate the findings in a supplementary file, adding a paragraph, say, to the results section to quote the findings.*

**RESPONSE:** We did an additional search for non-English articles using the same criteria from the original search and reviewed the translated abstracts. We identified 42 additional articles which were all screened and were all excluded as none met the inclusion criteria. The following sentence has been added to the methods and the results and Figure 1 has been updated to include the 42 additional articles. “We did another search on October 26, 2021 to update our systematic review to also include articles not in English from the same period of January 2000 to December 2020.”

1.2 *Please restructure the abstract: the current "Background" sentence should begin the "Methods and findings" subsection. We suggest crafting a new "Background" subsection of at least two sentences, aiming to explain why HIV prevention is important, for example, and the relevance of cash transfers in this area.*

**RESPONSE:** The abstract has been restructured to include the new background below.

“Background: Given the success of cash programs in improving health outcomes and addressing upstream drivers of HIV risk such as poverty and education, there has been an increasing interest in their potential to improve HIV prevention and care outcomes. Recent reviews have documented the impacts of structural interventions on HIV prevention, but broader evidence on cash transfer programs on HIV prevention has not been systematically reviewed for almost a decade. “

1.3 *Where you quote "45 publications" in the abstract, please add a few additional words to quote the number of studies screened and excluded, for example.*

**RESPONSE:** More detail has been added on the articles screened and included in full text review. “We screened 1565 studies and examined 78 in full text review to identify a total of 45 peer reviewed publications and reports from 27 different interventions or populations.”

1.4 *In the abstract, please make that "observational studies", "case-control study" etc, and make similar amendments in the results section (main text).*

**RESPONSE:** These edits have been made to the abstract and in the text.

1.5 *Please state the reason for not doing a meta-analysis in the abstract.*

**RESPONSE**: The following text has been added to the abstract. “We did not do a meta-analysis given the range of outcomes and types of cash transfer interventions assessed.”

1.6 *Where you refer to a "large effect of the program" in the abstract, for example, please confirm that this refers to evidence from randomized trials. We generally ask authors not to use language implying causality from weaker study designs.*

**RESPONSE**: The sentence has been edited to read “a large association between the program and sexual behaviors” given the inclusion of data from other types of studies. The term “effect” has been edited throughout to only refer to evidence from randomized trials.

1.7 *Regarding apparent program effects on sexual debut, different numbers seem to be quoted in the abstract ("10/18" studies), author summary ("11/18") and main text (line 185; "9/18"). Please check the numbers and report consistent findings throughout.*

**RESPONSE**: The number has been corrected to 10/18 throughout the manuscript.

1.8 *Early in the author summary we suggest "... a ... strategy".*

**RESPONSE***:* This has been edited to read *“*a widely used policy strategy”

1.9 *We think that should be "December 2020" in the author summary.*

**RESPONSE**: This has been corrected to December 2020.

1.10 *At line 76, please state the year for "February 4th".*

**RESPONSE***:* The sentence has been edited to read “February 4^th^, 2019”.

1.11 *Should "that" be removed at line 105?*

**RESPONSE**: “That” has been removed from the sentence. The sentence now reads “A total of 21 studies included a cash transfer intervention (33 publications or reports) (Table 1).”

1.12 *At line 116, should that be "HIV incidence or prevalence ..."?*

**RESPONSE**: “HIV incidence or prevalence” has been added to the sentence. “Eight studies examined HIV incidence or prevalence as an outcome and results were mixed.”

1.13 *Noting "p<0.05" at line 121, please quote exact p values where available or, for smaller values, "p<0.001" throughout the ms.*

**RESPONSE:** Exact p values have been added at line 121 and throughout the results section where available.

1.14 *At line 132, please amend the wording to "... found no significant effect on HIV prevalence ... although there was a non-significant reduction in one study" or similar*

**RESPONSE:** The sentence has been edited to the following **“**found no significant effect on HIV prevalence, although there was a non-significant reduction in one study”

1.15 *At line 143, please amend the current wording ("... smaller effect on syphilis") to indicate that the apparent reduction was not statistically significant (e.g., "... reduced prevalence of HSV-2 but not of syphilis.").*

**RESPONSE:** The sentence has been edited to **“**the other cash transfer trial conditional on school attendance among girls in Malawi reduced prevalence of HSV-2 but not of syphilis”

1.16 *At line 159 and any other instances in the ms, please substitute "sex" for "gender" where appropriate*

**RESPONSE:** “Biological sex” has been substituted for gender where appropriate throughout the manuscript.

1.17 *Again at line 170 (RR 0.86, 95% CI 0.67-1.1) please amend the wording to indicate more clearly that the apparent effect was not statistically significant.*

**RESPONSE**: The sentence has been edited to indicate that the effect was not statistically significant. “The South African cash transfer intervention conditional on schooling reduced the risk of having any sex partner in the last 12 months (RR 0.90, 95% CI: 0.83–0.99), but did not significantly reduce having more than one sex partner in the last 12 months (RR 0·86 (95% CI: 0·67 - 1·1).”

1.18 *At line 182, can you add a few words to give the reader an idea what "larger reduction" was investigated in the study/ies in question?*

**RESPONSE***:* The word “larger” has been removed from the sentence*. “*However, a similar cash transfer conditional on school attendance among young women in South Africa did not identify a reduction in partner age differences (RR 0.90 (95% CI: 0.72 – 1.12) or transactional sex (RR 0.95; 95% CI: 0.78 – 1.15”

1.19 *At lines 204-6, where you discuss the findings of an observational study, please adapt the language to avoid implying causality, e.g., "... HIV risk behavior were lower than with receipt of cash alone."*

**RESPONSE***:* We have edited the sentence to avoid causal language*. “…*was associated with a larger reduction in HIV-risk behavior than with receipt of cash alone.”

1.20 *At line 215, please make that "In this systematic review ...".*

**RESPONSE:** The sentence has been edited to the following: “In this systematic review of cash transfer programs for HIV prevention…”

1.21 *At line 243, please avoid "incredibly".*

**RESPONSE**: The word” incredibly” has been removed from the sentence.

1.22 *At line 287, should that be "we were unable ..."?*

**RESPONSE**: The sentence has been edited to read: “Given that we were unable to do a meta-analysis….”

1.23 *Please use the general style "... aged 18 years" throughout.*

**RESPONSE:** The manuscript has been edited to use the general style “…aged 18 years” throughout.

1.24 *We suggest hyphenating "cash-transfer" throughout when used as an adjective.*

**RESPONSE**: “Cash-transfer” has been hyphenated throughout when used as an adjective. For example, cash-transfer intervention and cash-transfer program.

1.25 *Please use the journal name abbreviation "PLoS ONE" in the reference list.*

**RESPONSE**: The journal name has been abbreviated to “PLoS ONE” in the reference list.

1.26 *Please provide additional information for reference 69. Is a URL available, for example?*

**RESPONSE**: The website and organization name have now been added to the reference.

**Reviewer #2:**

*2.1 The authors continue to be reluctant to include studies not in English. They might be interested in the article by Jackson, J L and colleagues "The accuracy of Google Translate for abstracting data from non--English--language trials for systematic reviews." Annals of Internal Medicine 2019 (171) 677-679. Not perfect but better than dropping them altogether.*

**RESPONSE:** Please see response to 1.1. We did an additional search for studies not in English and reviewed the translated abstracts. 42 articles were identified, and none met the inclusion criteria for the review.
