# Supplementary material for: Cash transfers for HIV prevention: A systematic review
Source: PLoS Med. 2021 Nov 29;18(11):e1003866. doi: 10.1371/journal.pmed.1003866 (PMC8668130; doi:10.1371/journal.pmed.1003866)
Supplement: Attachment — Submitted filename: Requests from the Editors and Responses.docx [file pmed.1003866.s010.docx]

**Requests from the Editors and Responses**

**Requests from Editors:**

1. Please avoid "almost a decade" in the abstract. We suggest "several years".

**RESPONSE**: The sentence has been edited in the abstract as follows. “*Recent reviews have documented the impacts of structural interventions on HIV prevention, but evidence about the effects of cash transfer programs on HIV prevention has not been systematically reviewed for several years.”*

2. We suggest quoting some additional findings from the study in the abstract: the observations in the two summary points beginning "Eight of the 27 studies ..." and "Of the studies ..." could be included (bearing in mind that the abstract will appear in Pubmed, whereas the Summary points will not).

**RESPONSE**: Thank you for noting that the abstract will appear in Pubmed, whereas the summary points will not. The two additional points that you mention have been moved from the summary to the abstract.

3. Once this is done, please adapt the summary points to avoid repetition.

**RESPONSE**: The points mentioned above have now been edited in the summary to avoid repetition.

**Requests from Academic editor:**

An issue that needs to be resolved is the inconsistency in the number of studies included / excluded.

1. There is some inconsistency in the number of studies included - Line 254 and 264 refers to 25 studies included in this review but the abstract gives 27 studies. I think it should be 27 – table 1 lists 27 studies.

**RESPONSE:** Thank you for catching this error**.** This was a typo and has now been fixed at line 254 and line 264. The correct number of studies is 27 studies.

2. The numbers in the consort don’t quite add up – In figure 1 there are 78 studies included and then 23 are excluded – that would mean there were 55 studies remaining but the consort shows 45. In the text it states “78 studies were included and 45 were excluded that did not meet inclusion criteria. We identified a total of 45 peer reviewed publications” – if 45 were excluded then it would leave 33 publications not 45.

**RESPONSE:** Thank you for catching this error. We have now double checked the numbers in figure 1. There was a typo in the number of ongoing studies which has now been corrected from 2 to 12 in figure 1. 78 studies were included in qualitative synthesis minus 21 systematic reviews or commentaries, and 12 ongoing studies=45 studies included. The text at line 75 has been corrected to read “*78 studies were included and 33 were excluded that did not meet inclusion criteria. We identified a total of 45 peer reviewed publications and reports from 27 different interventions or populations.”*

Minor comments –

3. authors should not refer to Table 1 in the abstract

**RESPONSE:** The reference to table 1 has been removed from the abstract.

4. extra full stop in line 223

**RESPONSE:** The extra full stop has been removed at line 223.

5. Figure 1 – the reason for excluding the 1484 papers should be provided. Does the 1642 include the non-English studies?

**RESPONSE:** A footnote has been added to the figure to state that the 1642 includes English and non-English studies. A second footnote has been added to state the reasons for excluding the 1484 papers. We did not originally provide this detail for studies that were screened because we used the PRISMA flow chart template.
